# Ostology and Surgery

**Published:** 1876-02

**Authors:** 


### II. OTOLOGY AND SURGERY.

1. *A New Method of Inflating the Middle Ear.* JOS. GRUBER. (*Allg. Wiener Med. Zeitung*, 1875, No. 42.)

In order to force air into the Eustachian tubes without using a catheter, the upper pharyngeal space must be tightly closed against the fauces. This is accomplished in the simplest way by the act of deglutition as shown by Politzer's method. *But the same can be achieved also by simply crowding the root of the tongue against the posterior portion of the palate while a forcible expiration is made simultaneously.* During this procedure the root of the tongue shuts off the cavity of the mouth against the fauces and pushes the soft palate backward and upward, and the simultaneously expired air raises the soft palate so much that it forms a complete diaphragm between the fauces and the space above the velum palati. The expired air, therefore, escapes neither through the mouth nor through the nose, as one can readily prove by holding a light before the nose and mouth; it will not be moved by an air current as it would be if the air passed out of these orifices. Even an unintelligent person can carry out the desired movements of the tongue and velum palati by pronouncing such monosyllables as hack, hick, hock, huck. And by holding the tongue in the position which is necessary for the pronunciation of ck, (or kk), the patient can, at his pleasure, keep the naso-pharyngeal space closed against the fauces for some minutes. This new method is executed like Politzer's : when the ivory tip of the air bag is inserted into one nostril and the nose hermetically closed around it with the fingers of one hand, the patient is directed to pronounce one of the above syllables while the bag is emptied. The advantages Gruber claims over Politzer's for his method are:

1. It does away with the swallowing of water against which many patients have a great antipathy (!), especially because they dislike to drink out of a glass which was used by other persons.
2. One can force the air into the middle ear for some time in succession, because the patient can prolong the pronunciation of kk.
3. One can auscultate the middle ear.

2. *Styptic Cotton in the Treatment of Otorrhœa.* THOS. G. MORTON. (*Philad. Med. Times*, 1875, No. 205.)

Encouraged by the success Morton obtained in a case with the styptic cotton about two years ago, he has since used no other treatment in a large number of cases of otorrhœa, and always with the same excellent result. Before using the cotton, the ear should be thoroughly cleansed with tepid water and the canal then wiped out; then a portion of the styptic cotton (large enough to fill the meatus) is carried in until the plug rests close to, but is not in contact with, the drum. The first plugs should be changed daily, then less frequently; no pain arises from the use of this preparation. As styptic cotton becomes friable and breaks up after a few months' exposure, it should be made fresh from time to time.

3. *Salicylic Acid in Aural Discharges.* J. J. CHISOLM, M.D. (*Western Lancet*, 1875, No. X.)

Seeing reports of the advantages derived from sprinkling powdered salicylic acid upon the surface of chronic ulcers, it was inferred that this new disinfectant may prove of value in aural surgery. Dr. C.'s experience was chiefly with the acid in powder diluted with calcined magnesia or oxide of zinc; though the pure acid could be very safely applied to the discharging tympanic membrane. He used one part of salicylic acid to two parts of magnesia. The ear being thoroughly cleansed, the powder was blown into the ear through a quill or a laryngeal powder-blower. The effect in all cases has been good; its controlling influence being much more marked in some persons than in others. In most cases the application has to be repeated daily before the aural discharge is stopped.

4. *Salicylic Acid as a Dressing for Wounds.* (*Philadelphia Med. and Surg. Rep.*, Jan. 1.)

At a society meeting in London, lately, Mr. Callender brought forward a series of cases illustrating the use of this agent as an application to wounds. The following preparations were chiefly employed : (*a*) Phosphate of soda, three parts ; salicylic acid, one part; water, fifty parts. (*b*) Salicylic acid, one part ; olive oil, forty-nine parts. (*c*) Salicylic acid, one part ; bicarbonate of soda, half a part ; water, 100 parts. Summing up his observations, Mr. Callender arrived at the following conclusions : Putting together its good points, he found that salicylic acid was free from odor, and so far was acceptable to the patients; that wounds healed under its influence, and, during the progress of repair, were free from bad smells; that, unless strong with spirit, or but little diluted, it did not cause local pain. Its bad points seemed to be these: That, above the strength of two per cent., it caused local irritation, with some constitutional disturbance ; and if the patient had a delicate skin, even the weak preparation was a source of trouble ; that there was more discharge from a wound dressed with salicylic than there was when carbolic acid was used; that its influence upon a recent wound, as after an operation, was not efficacious against the occurrence of decomposition as was that of carbolic acid, chloride of zinc, or of boracic acid; that the repair of a wound was less active, and the granulations, if any, more flabby than when other simple or antiseptic dressings were employed. On the whole, while admitting its use as a local application to be fairly commendable, Mr. Callender thought it inferior in its antiseptic properties to other agents, and did not consider it to be a remedy meriting the strong recommendations which had been given it by some of those who had made trial of it.

5. *Injections of Carbolic Acid in Poisonous Wounds.* KLAMANN and DORING. (*Allg. Med. Centralz.*, 1875, Nos. 99 and 101.)

Klamann reports the following case: On July 9, a young man who was stung in the nose by an insect, called on him with œdema of the right eyelids, and considerable swelling of the right side of the nose. The use of an ointment of carbolic acid did not arrest the inflammation, and by the next evening the whole right half of the patient's face was frightfully swollen. About fifteen minims (one gramme) of a two per cent. solution of carbolic acid was injected by an hypodermic syringe at the little red point which indicated the bite of the insect. The patient almost fainted, but the effect of the injection was quick. Within twenty-four hours the œdema disappeared and the patient was completely cured.

In Doring's case the œdematous infiltration had extended from the fingers to the shoulder in twenty-four hours after an insect had deposited some poisonous matter in a recent scratch of the skin at the dorsal side of the right hand. The skin of the swollen arm was hot, purple, and in three places it showed a dusky color. The patient, a robust man, was quite feverish. Five hypodermic injections, of fifteen minims each, of a two per cent. solution of carbolic acid, were made—three injections into those discolored spots and two above the line of œdematous infiltration into the healthy tissues. In the afternoon the injections were repeated, and the arm was dressed with cold water compresses during the night. On the 17th, patient felt better; the redness and swelling of the arm had decreased in intensity; the dusky spots had disappeared; four subcutaneous injections. And the following day the arm was all right.

6. *Sutures of Tendons.* TILLAUX. (*Centralb.*, 1875, No. 2.)

The tendons of the extensor of the little and ring fingers were torn by a hook; the wound healed, but one month later the two fingers were bent into the palm of the hand and could not be used at all. By the use of Esmarch's bandage, Tillaux tried to search for the tendons, but he could find the peripheric ends only. Exposing the tendon of the middle finger, he made a buttonhole in it, put the peripheric ends of the tendon of the little and ring fingers through it and fastened them together by a metallic suture. Extension and flexion of both fingers were completely restored by this operation.

7. *Removal of the Anterior Five Tarsal Bones and the Heads of the Metatarsal Bones.* W. W. DAWSON. (*Cincinnati Clinic*, 1876, No. 1.)

This operation was first performed by P. H. Watson, who gave an account of it in the *Edinburgh Med. Journal*, April, 1874. It is effected by making an incision on the inner and outer sides of the foot, between three and four inches in length; dissecting the soft parts off the dorsal and plantar surfaces of the tarsus, from the outer and inner sides, until the whole extent of the osseous tissues to be removed is deprived of its soft coverings; the diseased spongy bones, then, are removed by disarticulation and the use of a key-hole saw. Dawson performed this operation successfully on a little girl, æt. 6 years, in November, 1874. About eighteen months previous to the operation she was struck on the dorsum of the right foot. The blow did not seem to be a hard one, but violent inflammation followed it, involving first the soft parts and passing thence to the tarsus. In July, 1874, an abscess was formed on the inner border of the foot; when it was opened the tarsus was found in a carious condition. Another abscess was formed, to the distal side of the first, and the junction between the tarsus and metatarsus was found to be involved in the disease. The suffering of the child was very great; the pain at night unusually severe, and the foot exceedingly sensitive. Watson's tunneling operation was performed, the surgeon following his directions very closely; and by the use of Esmarch's bandage there was not an ounce of blood lost. The result has been most satisfactory; with her shortened foot the girl walks without a limp.

8. *Treatment of Varicocele and Attendant Irritability of the Genital Apparatus by Compression.* Dr. RAVOTH. (*Berl. Klin. Wochenschr.*, 1875.)

The apparatus employed by Dr. Ravoth is a combination of the suspensory bandage with an additional appliance for producing pressure, which differs from the apparatus hitherto in use and which has been condemned by Mr. Syme and others, in that it affords an easy and regular compression. It is claimed that without irritating, it accelerates the circulation and restricts the determination of blood to the parts involved, increases the tone of the vein and *cremaster*, and empties the vein by compression over the regurgitating blood column.

The first case cited, and the only one which for the sake of illustration may be cited here, is one of a number treated successfully by the method under consideration.

X., a student, age 21, suffers from a left-side varicocele, which dates back to his 16th year. He is unable to assign a cause. No evidence of hereditary predisposition. Has never been guilty of onanism. Three years ago emissions became frequent, the number of which gradually increased until, at last, they occurred, at least, nightly, often even twice in the night and during the day, also, without sexual desire or erection. There was a certain rudeness of demeanor and permanent despondency of mind. Patient of medium size, not of weakly habit, pale, expression dejected, and apparently older than he is. Appetite voracious, digestion, with the exception of sluggishness at stool, undisturbed. The varicocele is fully developed. The scrotum hangs loose and flabby, despite a suspensory bandage; measures 8 ctms. from the ring to the left testicle. Superficial veins are not dilated, neither is the spermatic cord. On the contrary, the left *venæ spermaticæ* have become considerably lengthened and form a cylindrical convolution around the testicle, atrophied to the size of a pigeon's egg. Upon pushing the left testicle somewhat upwards, the circumference was measured and found to be 12 ctms. The convolution has a flabby, doughy feel, but the veins may be easily distinguished from the *vas deferens.* No pain of any kind. Patient was under my care from June 17 to Oct. 19—four consecutive months. The treatment was begun with the application of the pressure-appliance in combination with the *suspensorium*, June 17th. During the first few days he had to learn how to manipulate and regulate the compressor. He increased the pressure by means of a *ratchet* two to three hours daily, and took it off altogether during the night. On July 2nd, there was a remarkable improvement. The length of the varicocele was only 6, and the greatest circumference 10½ ctms. But two nocturnal emissions had taken place in the last eight days, and the diurnal waste had ceased altogether. July 8th, patient in great distress of mind; complained of a return of the emissions, and was directed thereupon to have his back rubbed morning and evening with a liniment composed of tinct. canth., 5.0; sp. form. 150.0, (this has been found also very useful in enuresis noct.), and to take drops consisting of acid. phosph. and aq. amygdal., *aa* 20—25 *ter die*, and at bedtime a powder of lupulin, 0.3; camphor, 0.03. July 28, substantial improvement. Length of varicocele, 5; circumference, 10 ctms. Both nocturnal and diurnal emissions ceased. Much improved in spirits. From this time on all other means were discontinued, the patient wearing the compressor only. Oct. 19, saw him for the last time. Was very well; rode horseback daily without any aggravation or increase of the varicocele, which immediately after a brisk ride measured 5 ctms. in length and 10 ctms. in circumference. The left testicle equaled the right in size and other qualities. The emissions have not returned.

In the remaining cases cited in this lecture, the best results followed upon the use of the compressor and *suspensorium*, without collateral measures.

Several English surgeons have recommended this method of treatment and reported favorably, to wit: Curling (*Lond. Med. and Surg. Trans.*, xxix, p. 259), Morton (*Dub. Quart. Jour. of Med. Sci.*, 1851, p. 319), and Thompson (*Monthly Jour. and Retrosp. of Med. Sci.*, No. xix, November, 1848).

It is remarkable that these surgeons speak only of varicocele, while the secondary irritability is mentioned only by Morton.

Concerning the comparative frequency of varicocele the subjoined statistics are interesting and significant. Of 6,025 rejected, out of 17,540 enlisted in 1844, 229 were rejected for hernia, 424 for varicocele and 582 for varix. In 1845, of 13,370 enlisted, 4,146 were rejected, of which, 264 for varicocele and 227 for hernia. Of 140 cases of varicocele, 122 were left side, 6 right side and 2 double. Dr. Ravoth's observation yields 2 per cent. recti-lateral and 2 per cent. bilateral. These statistics show plainly the demand upon our attention, to which these affections are entitled.

Slavjansky has accomplished remarkable results by exciting the N. splanchnici by electricity, creating an acceleration of circulation in the cava, (*Arbeiten aus dem Physiol. Institut in Leipsig*, 1874). Though electricity and compression are widely different agents, there is yet a certain analogy in their *modus operandi.*

